# Experiences of armed conflicts and forced migration among women from countries in the Middle East, Balkans, and Africa: a systematic review of qualitative studies

**DOI:** 10.1186/s13031-022-00481-x

**Published:** 2022-09-07

**Authors:** Linda Jolof, Patricia Rocca, Monir Mazaheri, Leah Okenwa Emegwa, Tommy Carlsson

**Affiliations:** 1 The Red Cross Treatment Center for Persons Affected By War and Torture, Malmö, Sweden; 2 Department of Nursing Science, Sophiahemmet University, Stockholm, Sweden; 3 Department of Neurobiology, Care Sciences, and Society, Karolinska Institutet, Huddinge, Sweden; 4 The Department of Health Sciences, The Swedish Red Cross University, Huddinge, Sweden; 5 The Department of Women’s and Children’s Health, Uppsala University, Uppsala, Sweden

**Keywords:** Armed conflicts, Internal displacement, Life change events, Qualitative research, Refugees, Systematic review, Women

## Abstract

**Background:**

A significant proportion of the global population is displaced, many being women. Qualitative studies can generate in-depth findings that will contribute to an understanding of their experiences, but there is a need for further synthetization efforts. The aim was to provide a comprehensive perspective about adult women’s experiences of armed conflicts and forced migration, focusing on women in or from countries in the Middle East, Balkans, or Africa.

**Methods:**

Systematic review of English reports presenting empirical qualitative studies published in scientific journals 1980 or later, utilizing searches performed in September 2021 within three databases combined with manual screening. Of the 3 800 records screened in total, 26 were included. Methodological details and quality were appraised using pre-specified extraction and appraisal tools. The findings within the included reports were analyzed with thematic analysis.

**Results:**

Most reports utilized interviews, including in total 494 participants, and were appraised as having insignificant methodological limitations. The first theme concerns changed living conditions, involving reduced safety, insufficient access to resources meeting basic needs, forced migration as a last resort, and some positive effects. The second theme concerns the experienced health-related consequences, involving psychological distress, risks during pregnancy and childbirth, exposure to violence and discrimination as a woman, as well as a lack of adequate healthcare services and social support. The third theme concerns the resources and strategies that enhance resilience, involving social support and family life, as well as utilization of internal resources and strategies.

**Conclusion:**

When experiencing armed conflicts and forced migration, women face significant challenges related to changed living conditions and are exposed to health-related consequences. Consistently, women are targets of severe structural and personal violence, while lacking access to even the most basic healthcare services. Despite facing considerable hardships, these women display extraordinary resilience and endurance by finding strength through social support and internal resources. Synthesized qualitative research illustrates that women value social support, including peer support, which is a promising intervention that needs to be evaluated in future experimental studies.

**Supplementary Information:**

The online version contains supplementary material available at 10.1186/s13031-022-00481-x.

## Introduction

Armed conflicts, defined as organized violence between state/s and/or non-state parties leading to fatalities within a population, are a major global concern with a continued high prevalence [1]. People who live in areas where armed conflicts occur are exposed to a wide range of violent actions and violations of human rights, including forced displacement, gunfire, shelling, and torture [2]. In addition to the severe impact it has on an individual level, political violence also involves large-scale impacts on communities and governmental functioning, including the destruction and control of public spaces as well as the deterioration of social systems such as healthcare services [3]. When faced with the significant dangers and ongoing adversities associated with armed conflicts, many have no other option than to leave their homes and take flight. Indeed, a significant proportion of the global population—in 2020 over 82 million individuals—are forcibly displaced as refugees, asylum seekers and internally displaced persons [4]. Approximately half of all displaced persons are women, many of these originating from countries in the Middle East, Balkans, and Africa. According to the UNHCR, a significant proportion of refugees originate from just ten countries in the world, eight being countries in these specific geographical areas [4].

Violence against women is an extensive global public crisis permeating socio-demographic variables and transcending national borders [5]. Displaced women and women living in the aftermath of an armed conflict are at an increased risk of experiencing physical, mental, and sexual violence [6–12]. When exposed to armed conflicts and/or forced migration, women suffer a wide range of severe short- and long-term health-related physical and psychological consequences. Repeatedly and uniformly, studies show a high risk of mental health burdens among refugee women, including symptoms of posttraumatic stress and depression [13–17]. Refugees also show a high prevalence of various serious non-communicable and communicable diseases, including diabetes, hypertension, and HIV [17–20]. Obstetric complications are also common, with higher rates of maternal and neonatal morbidity among forced migrants compared with non-migrant populations [17, 21–24]. Further complicating their health-related situation, reports suggest that women refugees experience unmet health needs and suffer structural inequalities in access to healthcare services [19], including reproductive health services [23, 25, 26]. Taken together, research indicates a situation where refugee women experience significant health-related consequences and mental health burdens when exposed to armed conflict, torture and/or forced migration.

In addition to focusing on the burdens and distress among refugees, the intrapersonal and interpersonal conditions that can strengthen their ability to cope and enhance their health has gained increased attention in research. Broadly defined as the capacity to bounce back or recover from stressful or traumatic events [27], resilience in refugees is indeed suggested to be associated with improved mental health [28]. The concept is multidimensional, involving both internal and external protective factors [27]. One previous review highlights the importance of cultural, social, material, and personal factors that promotes resilience in refugee women who are resettling in a host country. However, the same review calls attention to the general underrepresentation of refugee women in research and the need for further synthesis of qualitative evidence [29].

Qualitative studies are research endeavors that can be utilized to explore lived experiences and generate in-depth findings that will contribute to a holistic understanding of human suffering. In recent years, there has been a steady growth in qualitative research exploring lived experiences among women who are forced migrants. However, much of this research focuses on narrow topics, often surrounding reproductive health among women who are resettling in the host country. Reviews investigating qualitative research about refugee women’s post-migration experiences have revealed various challenges they encounter, as well as factors promoting resilience, while resettling in the host country [29–31]. To the extent of our knowledge, less synthetization efforts have however been made regarding women’s experiences before the resettlement. Moreover, displaced persons are a heterogeneous population constituting of a wide range of personal characteristics, including country of origin. To reach clinically applicable results about a defined group of women, this review focuses on women originating from countries currently commonly represented among refugees in European countries. Thus, the aim of this systematic review was to provide a comprehensive perspective on qualitative research about adult women’s experiences of armed conflicts and forced migration—focusing on women in and from the Middle East, Balkans, or Africa.

## Methods

### Design

This was a systematic review of reports presenting empirical qualitative studies published in scientific journals. This review is reported according to the Enhancing Transparency In Reporting The Synthesis Of Qualitative Research (ENTREQ) guideline (Additional file 1: Table S1) [32]. A qualitative systematic review is appropriate when the purpose is to provide overarching summaries and integrations of qualitative studies exploring lived experiences [33].

### Search methods

Pre-planned systematic searches were performed in September 2021 utilizing the three databases CINAHL, PsycINFO, and PubMed. Through discussion and pilot searches, relevant search terms were identified. Boolean operators were utilized to form the final search string “woman AND (experience OR perception OR understanding) AND (war OR armed conflict OR warfare OR forced migration OR forced displacement OR torture)”. To identify further reports, manual screening was also performed, by inspecting the reference lists in the included reports and by searching through the lists of citations in the databases.

### Eligibility criteria

To be included, reports needed to meet the following criteria: (1) present qualitative findings from an empirical study; (2) written in English; (3) published 1980 or later; (4) include findings based on women as the primary source of information; (5) only having included women in or from countries in the Middle East (herein defined as also including Afghanistan, while we acknowledge that this country is not always considered part of the Middle East), Balkans, or Africa; (6) based on primary qualitative research published as an article in a scientific journal; and (7) having clearly distinguishable results speaking about the pre-migration and/or peri-migration experiences among women. Reports were excluded if: (1) only reporting findings about post-migration experiences; (2) including findings based on secondary sources; and (3) lacking full-text documents (Table 1). No studies were excluded based on methodological quality.

**Table 1:** Inclusion and exclusion criteria

Domain	Inclusion critera	Exclusion criteria
Population	Adult women (18 years or older) who have experiences of war, torture, and/or forced migration (internally displaced, forced migration between countries, or living in refugee camps), in or from countries in the Middle East, Balkans, or Africa	Non-forced migrants; secondary sources of qualitative data; Women in or from countries in other regions in the world; Other genders than being a woman
Phenomenon	Experiences of war, torture, and/or forced migration	Only reporting about post-migration experiences
Language	English	Non-English
Study design	Qualitative research	Quantitative research; Mixed- or multi-methods research
Publication time	Published 1980 or later	Published before 1980
Publication type	Primary research published as article in scientific journal	Conference proceedings or abstracts; Book chapters; Literature reviews; Letters/Editorials; Commentaries; No full-text document; Theses

### Study selection

The first two authors performed the screening procedure independently. Initially, all titles and abstracts were screened for inclusion and marked as included, excluded, or ambiguous. They discussed their screenings and all reports with conflicting decisions or still marked as to maybe include after discussion were carried over to the next step in the screening process. All reports remaining after this initial screening were extracted as full-text documents and read by the first authors to assess final eligibility. Ambiguous cases were discussed with the last author until consensus was reached.

### Search outcomes

**Fig. 1:**
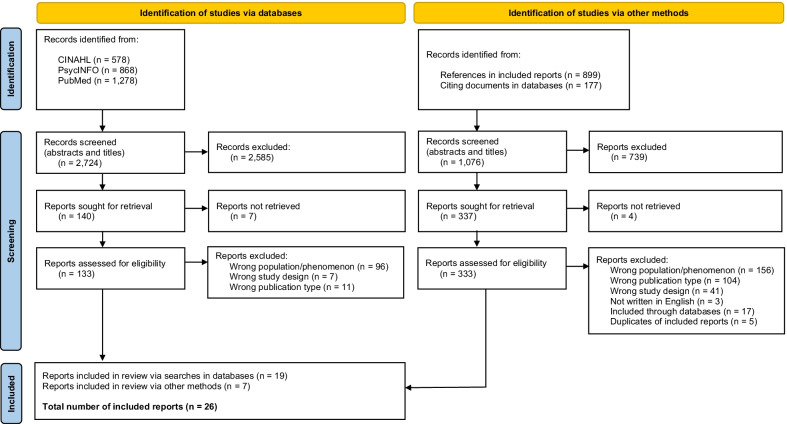
The process of searching and screening for reports

In total, the systematic searches yielded 2 724 hits. Among these, 2 585 were excluded based on the screening of titles and abstracts, and seven were inaccessible, resulting in 133 reports read as full-text documents. A total of 114 reports were excluded in this phase, because of the population or phenomenon investigated (n = 96), publication type (n = 11), and study design (n = 7). Thus, 19 reports identified via systematic searches in databases were included. Manual searches in reference lists and through citing documents (empirical studies published in scientific journals) in databases resulted in another 1 076 reports identified. Among these, 739 were excluded based on irrelevance or the methodological characteristics conveyed in the titles, leaving 337 more closely inspected. Four reports were inaccessible and the remaining reports were excluded because of the population or phenomenon investigated (n = 156), publication type (n = 104), study design (n = 41), and not written in English (n = 3). After removing duplicates and previously identified reports, a total of seven were included through manual searches. This resulted in 26 included reports in total within this review (Fig. 1).

### Data extraction and quality appraisal

Methodological details were jointly extracted from all reports by the first two authors and the last author utilizing a pre-designed tool, including details about the: (1) date of primary study; (2) purpose; (3) population under study; (4) sample size; (5) methods for data collection; and (6) data analysis. The methodological quality of the included publications was appraised using the appraisal tool developed by The Swedish Agency for Health and Technology Assessment and Assessment of Social Services [34]. The appraisal instrument included five areas: (1) underlining theory, model, or theoretical framework; (2) participants; (3) data collection; (4) analysis; and (5) researcher’s role. The first two authors conducted joint appraisals of the included reports, and the last author scrutinized all their appraisals after reading all included reports. Disagreements were settled through discussions among the first two and the last authors.

### Data abstraction and synthesis

Sections relating to the qualitative findings in each of the included reports were extracted and analyzed with thematic synthesis, a flexible and useful approach to synthesize qualitative material depicting lived experiences [35]. The analysis process consisted of six phases, and an inductive approach was chosen to remain open towards the findings presented in the reports. First, familiarization with the data was achieved through immersion and repeated reading. Second, initial codes were manually generated through a systematic process involving the extraction of relevant and interesting chunks in the findings of the reports. Third, codes were collated into descriptive themes and sub-themes, defined as a patterned meaning that has relevance for the aim of the study. The later stages aimed to generate analytical themes that go beyond the content of the included empirical studies. These steps were inspired by the thematic approach to empirical studies as depicted by Braun and Clarke [36]. The themes were refined to ensure adequate levels of internal and external consistency, leading to the construction of a thematic map detailing the findings depicting analytical themes. The identified themes in the thematic map were further defined and labeled, resulting in a detailed description of the content within each of the analytical themes. Lastly, the findings were produced in full, compiling comprehensive final summaries of the themes and identifying illustrative examples extracted from the reports. The first two authors were responsible for the primary analysis, conducting all steps separately and then discussing their findings until consensus was reached among them. The remaining authors provided feedback at various stages of the analysis, all senior researchers with experience conducting qualitative analyses.

## Findings

### Methodological characteristics and quality appraisal

The methodological characteristics of the reports are presented in Table 2. The included reports were published between 2005 and 2021, with 12 being published 2019 or later. The utilized methods for data collection involved interviews (n = 16), focus group discussions (n = 3), observations (n = 3), narrative diaries (n = 1), and narrative timelines (n = 1), with five studies utilizing more than one data collection method. Participant recruitment included purposeful (n = 6), convenience (n = 2), snowball (n = 1), a combination of purposeful and snowball (n = 1), and a combination of convenience and snowball (n = 1) sampling. The methods of analysis included thematic (n = 5), content (n = 3), narrative (n = 3), phenomenological (n = 3), ethnographical (n = 2), and grounded theory/constant comparative (n = 2) analysis. Eight reports did not specify the method of recruitment and one report did not specify the method of analysis. The reports included participants from Palestine (n = 4), Somalia (n = 4), Afghanistan (n = 2), Bosnia (n = 2), Syria (n = 2), West Africa (n = 2), Ethiopia (n = 1), Ghana (n = 1), Nigeria (n = 1), and Rwanda (n = 1), in total 494 participants. When disclosed, the migration statuses of the participants were refugees or skilled/family reunification immigrants (n = 6), asylum seekers (n = 1), internally displaced (n = 1), having permanent residence permit in the host country (n = 1), and repatriated refugees (n = 1). Participant ages ranged from 18 to 80 years.

**Table 2:** Methodological characteristics of the included reports (n = 26)

Authors (year), region where conducted	Aim	Participant’s region of origin (n), recruitment	Data collection	Analysis	Grade
Al Issa et al. [57] (2021), Israel	Explore prevalence of, type of and reaction to sexual violence	Palestine (20), Purposeful	Interviews	GT/CC	**
Al-Natour et al.[42] (2019), Jordan	Describe lived experience of marital violence toward refugee women during war	Somalia (16), Purposeful	Interviews	PA	***
Al-Natour et al.[61] (2022), Jordan	Highlight experiences of the war–refugee families who have sought shelter in a host country	Somalia (16), Purposeful	Interviews	CA	***
Babatunde et al.[41] (2020), Australia	Highlight how past experiences of resilience and strength of migrant women can be vital in informing care provided by mental health practitioners	West Africa (22), Convenience and snowball	Interviews	TA	***
Byrskog et al.[39] (2014), Sweden	Explore experiences and perceptions on war, violence, and reproductive health before migration	Somalia (17), Purposeful	Interviews	TA	***
Hirsch et al. [48] (2012), Gaza	Trace mother’s own experiences, thoughts, and feelings after being exposed to rocket attacks	Israel (52), Convenience and snowball	Survey, interviews	CA, TA	**
Horn et al. [62] (2014), Sierra Leone and Liberia	Explore women’s perceptions of causes of intimate partner violence and the ways they understand these causes to interact with the experiences of war	West Africa (130), Not specified	Interviews, focus groups	TA	***
Lalla et al. [55] (2020), Kenya	Understand the ways women experienced insecurity at a refugee camp	Somalia and Ethiopia (20), Snowball	Interviews, observations	EA	***
Mannell et al. [50] (2021), Afghanistan	Explores women’s lived experiences of domestic violence and conflict	Afghanistan (20), Convenience	Interviews	TA	***
McGadney-Douglass et al. [38] (2008), Ghana	Not specified	Ghana (20), Convenience	Focus groups	Not specified	**
Mukamana et al. [58] (2008), Rwanda	Explore lived experience of women who were raped during genocide	Rwanda (7), Purposeful	Interviews	PA	***
Pavlish [59] (2005), Rwanda	Describe refugee women’s action responses to difficult living situations	Democratic Republic of the Congo (14), Purposeful	Interviews	NA	**
Rizkalla et al. [56] (2020), Jordan	Psychological aim taking into account emotions, cognitions, and relational dynamics of refugee women with the aim of informing interventions and policies that advance refugee well-being	Syria (24), Not specified	Interviews	NA	***
Rizkalla et al. [45] (2021), Jordan	Examine refugee women’s experiences from the war’s outset through their journey and addresses the toll this journey had on their lives	Syria (24), Not specified	Interviews	NA	***
Robertson et al. [51] (2007), Bosnia	Describe displaced mothers’ experiences caring for their children during and immediately after war	Bosnia (14), Purposeful	Interviews, observations	EA	***
Ross-Sheriff [52] (2006), Afghanistan	Were women helpless victims or actors during war, in exile, and during repatriation to their homeland; what roles did the women play; what coping strategies did the women use	Afghanistan (60), Not specified	Interviews	GT/CC	**
Shehadeh et al. [49] (2016), Israel	What are the difficulties captives’ wives experience; what are the sources of support that these wives receive; how do they deal with these difficulties, and how do they cope with this situation	Palestine (16), Not specified	Interviews	TA	**
Sherwood et al. [46] (2012), United Kingdom	Explore women’s experiences of violence during conflict	Somalia and Zimbabwe (6), Purposeful	Interviews	GT/CC	***
Skjelsbaek [40] (2006), Not specified	Generate knowledge about war rape and show how women employ different strategies for war-rape survival and identity construction	Bosnia (5), Not specified	Interviews	NA	**
Sandole et al. [44] (2013), Rwanda	Understand the process by which wartime rape affected women’s sense of self and identity before, during, and after genocide	Rwanda (30), Not specified	Interviews	GT/CC	**
Sossou et al. [43] (2008), USA	Investigate personal lived experiences through the war and the resilience factors that have contributed to general well-being, despite traumatic experiences	Bosnia (7), Convenience	Interviews	TA	**
Sousa et al. [54] (2021), Gaza	Explore the shifting, unpredictable, and traumatic nature of life during a major military operation	Palestine (21), Purposeful and snowball	Diaries, interviews	CA	***
Sousa et al. [60] (2020), Palestine	Explore mothering and political violence	Palestine (32), Not specified	Focus groups, observations	CA	***
Tessitore et al.[53] (2021), Italy	Explore subjective meanings asylum seekers attribute to their pre-migratory, migratory and post-migratory experiences, with an examination of the gender identity dimensions	Nigeria (5), Not specified	Interviews	PA	***
Veronese et al. [47] (2021), Gaza	Investigate the consequences of war and political violence for women’s mental health and psychological functioning	Palestine (21), Purposeful and snowball	Interviews	TA	***
Veronese et al. [37] (2021), Gaza	Explore specific factors that contribute to women’s individual and collective perceptions about war and associated traumatic life events that occurred during their lives	Palestine (21), Not specified	Narrative timelines	TA	***

*CA* Content analysis, *GT/CC* Grounded theory/constant comparative method, *EA* Ethnographic analysis, *NA* Narrative analysis, *PA* Phenomenological analysis, *TA* Thematic analysis

**Moderate methodological limitations

***Insignificant or less methodological limitations

The methodological appraisal revealed that the majority of the included reports had acceptable, or unclear, methodological shortcomings (Table 3). More than 50% of the reports were appraised as not having serious shortcomings, including the coherence (n = 24, 92%), analysis (n = 22, 85%), participants (n = 20, 77%), data collection (n = 16, 61%), and researchers (n = 15, 58%). All reports were appraised as relevant. The overall methodological limitations according to the utilized tool were judged as insignificant or less (n = 17, 65%) and moderate (n = 9, 35%).

**Table 3:** Methodological appraisal of included reports (n = 26)

Topics/question	Yes (n)	No (n)	Unclear (n)
*Adherence between philosophical stance/theory and sample/methodology*			
Purpose and question related to theory or philosophical stance	25 (96%)	–	1 (4%)
*Participants*			
Sample appropriate to answer the question	24 (92%)	–	2 (8%)
Recruitment method appropriately chosen and implemented	13 (50%)	–	13 (50%)
Serious shortcomings affecting reliability	2 (8%)	20 (77%)	4 (15%)
*Data collection*			
Serious shortcomings in data collection affecting reliability	1 (4%)	16 (61%)	9 (35%)
*Analysis*			
Analysis appropriate and carried out in an appropriate manner	22 (85%)	–	4 (15%)
Researchers reflexive when interpreting data	7 (27%)	1 (4%)	18 (69%)
Interpretations validated	15 (58%)	5 (19%)	6 (23%)
Serious shortcomings in analysis affecting reliability	1 (4%)	22 (85%)	3 (11%)
*Researchers*			
Researchers have any relationship with the participants	1 (4%)	7 (27%)	18 (69%)
Researchers handled their preconceptions in an acceptable way	8 (31%)	1 (4%)	17 (65%)
Researchers independent of financial or others conditions	13 (50%)	1 (4%)	12 (46%)
Serious shortcomings affecting reliability	2 (8%)	15 (57%)	9 (35%)
*Coherence*			
Majority of the data used in the analysis	25 (96%)	–	1 (4%)
Conflicting data handled appropriately	–	–	26 (100%)
Collected data support the findings	25 (96%)	–	1 (4%)
Serious weaknesses that can lead to a lack of coherence	–	24 (92%)	2 (8%)
*Sufficient data*			
Number of participants large enough	22 (85%)	–	4 (15%)
Form of data collection allows opportunity for rich data	25 (96%)	–	1 (4%)

### Thematic analysis

The thematic synthesis resulted in three themes illustrating women’s lived experiences. The first theme, involving four sub-themes, concerns the changed living conditions presented to women when exposed to armed conflict and forced migration (Table 4). The second theme, involving five sub-themes, concerns the health-related consequences experienced by women when exposed to the complex and demanding situation (Table 5). The third theme, involving two sub-themes, concerns the resources and strategies that enhance resilience and strength in these women (Table 6).

**Table 4:** Summary of content in the identified sub-themes in the first theme

Sub-theme	Summary of content illustrating women’s experiences
Reduced safety and exposure to general violence	Life became unstable and unsafe, with increased risk of violence [37–50]
	Conflicts resulted in destruction of societal structures and relationships [40, 45–47, 49, 58, 59]
	Violence was expressed as threats, destruction, bombings, torture, sexual violence, theft, shootings, murders, obstacles to work, and arbitrary arrests [38–40, 45–49, 52–56]
	Killings, abuse, and violence against family members and neighbors were witnessed [39, 40, 46, 56, 60]
	Women suffered violence based on ethnicity and religion [40, 55, 57]
	Family relationships changed and family bonds were disrupted [37, 39–41, 44, 46–49, 57, 59]
Insufficient access to resources meeting basic needs	War reduced the access to resources needed to meet basic needs [42, 51, 54, 59, 60]
	Grief over being unable to provide resources, safety and optimism for children [39, 59, 60]
	Socioeconomic stress was experienced, including financial and housing difficulties [39, 42, 47, 54]
	When living in refugee camps, women experienced significant challenges, including food shortages, insanitary conditions and inadequate shelters [38, 41, 42, 45, 55, 61]
Forced migration as a last resort	Reasons for migration included: (i) a need to seek protection; (ii) a need to flee from destroyed structures; (iii) when experiencing a lack of resources, and (iv) wanting to seek out peace and freedom [38, 39, 41, 45, 52, 56]
	The decision to migrate: (i) was not easy but needed to keep the family safe and seek peace [39, 45]; (ii) involved having to renounce normality and property [38, 45]; and (iii) was taken through social support and some needed to persuade family members [38, 45]
Positive effects related to exposure	Responsibilities of family members were expanded, involving increased independence [46, 48, 52, 62]
	Relationships with family members were strengthened through the exposure to war [47, 48, 54]

**Table 5:** Summary of content in the identified sub-themes in the second theme

Sub-theme	Summary of content illustrating women’s experiences
Psychological distress and during migration	Legal and illegal migration routes involved fear and uncertainty among women [43, 45–48, 56, 59]
	Women were reminded about dangers when witnessing the death of people [53]
	Having to take responsibility of others during migration involved psychological distress [45, 51]
	Women placed in refugee camps experienced the setting as: (i) unsafe associated with significant suffering [42, 43, 45, 55, 59]; and (ii) inhospitable environments lacking basic facilities and involving poor living conditions [38, 41, 42, 45, 55, 61]
	Being forced to migrate involved feeling a loss of identity, difficulties accepting their identity, challenges when trying to adjust, and feeling grief or emptiness when missing and longing for their previous life and country of origin [43–46, 52, 56]
Being exposed to risks during pregnancy and childbirth	Pregnancy and childbirth involved a risk of violence, resulting in serious consequences [55]
	Severe violence and risks when pregnant led to feeling unsafe and taking precautions [55]
	Migration meant little possibilities to access adequate postpartum care [39, 51]
	Migration led mothers to discontinue breastfeeding and had difficulties feeding children [39, 51]
Being exposed to violence and discrimination as a woman	Women were exposed to intensified violence in public and private settings [38–40, 45, 46, 50, 54, 55, 61, 62]
	The regime, military, and civilians were perpetrators of violence against women [39, 40, 45]
	Violence resulted in fear, panic, grief, feeling unsafe, and physical consequences [37, 54, 55]
	Violence continued in refugee camps and during migration [45, 52, 53, 55, 56]
	Women were often exposed to forced marriage [39, 50, 62] and intimate partner violence [39, 50, 55, 61, 62]
	When their husband died, women faced dangers and reduced social possibilities [50, 51]
	Women were at risk of sexualized violence, including repeated rape [39–41, 44–46, 53, 55, 57–59, 61]
	When victims of sexual violence, women: (i) were often silenced, faced stigma, and experienced social exclusion [39, 40, 42, 44, 46, 55, 58]; and (ii) experienced limited possibilities to seek abortion and legal support [39]
	To reduce their risk of sexualized violence, women were isolated from society [38, 39, 42, 55, 61]
Lack of social support	Insufficient social support were experienced from relatives [52] and organizations [38, 44, 46, 49, 56]
	A lack of social support contributed to feeling alienated and isolated [42, 47]
	Women experienced refugee camp staff as not having the necessary resources to offer support and that their actions at times are a threat to women’s safety [55]
Lack of adequate healthcare services	Women experienced challenges accessing healthcare during pregnancy and childbirth [45, 55]
	Various structural barriers contributed to reduced access to healthcare services [55, 56]
	Women experienced insufficient access to necessary medications [56, 61]
	Women experienced disrespectful and unethical behavior among healthcare professionals [55]

**Table 6:** Summary of content in the identified sub-themes in the third theme

Sub-theme	Summary of content illustrating women’s experiences
Social support and family life	Social support, including peer support, was important before, during, and after forced migration, as women exchanged emotional, informational, and instrumental social support [37, 39, 41, 42, 46, 49, 52, 54, 59]
	The family was a source for enduring difficult experiences, providing relief and reducing psychological distress [37, 43, 46, 49, 52, 54, 56, 59]
	Motherhood in itself was a source for resilience and agency [39, 41, 46, 47, 49, 51, 53, 54, 59, 60]
	Social support was essential during stressful circumstances such as pregnancy and childbirth [39]
Utilizing internal sources and strategies	Women tried to uphold daily life and their safety, living day by day [37, 39, 48, 49, 54]
	Women were hopeful of a better future, for example through religion and faith [39, 41–43, 46–49, 52–54, 56, 57, 59]
	Women stopped visiting relatives in jail [57]
	Women became violent towards their children and developed self-harming behaviors [42]
	Women succumbed to passivity and resignation as a coping mechanism [48, 59]
	Women kept their feelings concealed, were vigilant, and acted like they didn’t understand [51, 52, 60]
	Women were resourceful in: (i) getting men to be less violent and to protect themselves and others [38, 42, 51, 59]; (ii) retaining mental stability and restarted their lives when necessary [41, 54]
	Women took control of the situation by finding ways to make a living and find safe places [39, 41, 46, 48, 51, 52, 55, 59]
	Engaging in political activism was empowering [48, 54]

#### Theme 1: changed living conditions involving exposure to considerable discrimination, violence, death and a need to survive on a daily basis while taking care of their family

##### Reduced safety and exposure to general violence

When war came, an often comfortable and peaceful life [37–44] changed to an unstable and unsafe situation [40–42, 44–49] involving immediate danger [47, 48, 50], reduced hope [37, 44], and forced migration through severe settings [40, 41, 45, 51–53]. The violence was expressed as threats, destructions, bombings, torture, sexual violence, thefts, shootings, murders, obstacles to work, and arbitrary arrests [38–40, 45–49, 52–56]. Sometimes, it was based on women’s ethnicity or religion [40, 55, 57]. Women witnessed the destruction of their society [45, 58, 59] and were forced to witness executions of people around them [40]. They also witnessed mass murder, abuse and sexual violence against family members and neighbors [39, 40, 46, 56, 60]. Sometimes, their political commitment led to friends deciding to take distance from them [45] or led them to being subjected to violence [46]. Being exposed to war meant changed or divided family relationships [47, 49], that the roles within the family and society changed [46, 49], and a risk of losing family members, leaving them with the responsibility of taking care of any surviving children in the family [37, 39–41, 44, 46–48, 57, 59, 60].

“She goes on to provide details of the first attack on her village; how she became separated from her family; how she saw family members, relatives and neighbours killed; and how she was taken to a house where she was kept prisoner” [40]

”We lost my younger brother during the war; he was about 6 years old. When the rebels attacked, everybody was trying to run into the bush; you see, my brother was very young and there were gunshots, everybody tries to run; mother, brother, sister. He was very young and small. So, he fell in front of the door and was caught by the rebels’ commando.” (Direct quote from a participant) [41]

##### Insufficient access to resources meeting basic needs

Armed conflict and destroyed infrastructure hindered access to basic commodities such as food, water, electricity and fuel [51, 54, 59–61]. The lack of resources and options resulted in a grief of not being able to provide their children more than absolutely necessary [39, 59]. Women felt sad and powerless when seeing their children fearful and losing their innocence, while not to being able to convey optimism and safety [60]. Being exposed to war also entailed a significant socio-economic stress involving unemployment, difficulties earning a living, and inability to maintain the previous lifestyle [39, 47, 52, 56, 61]. Women lived in crowded, temporary, and unsafe housing situations [47, 54]. When living in refugee camps, women experienced significant challenges, including food shortages, insanitary conditions and inadequate shelters [38, 41, 42, 45, 55, 61].

“Women’s narratives about powerlessness in terms of not being able to meet their children’s basic requirements, particularly for education and healthcare, reflected considerable psychological pain” [60]

”I have to feed them... I have to find a way... no matter how bad the conditions are... Oh, I had a hard time... there was big hunger. We were eating the grass... many things... eating whatever you find just to survive. Leaves from the trees, we were boiling and eating.” (Direct quote from a participant) [51]

##### Forced migration as a last resort

When all other options failed, women were forced to take the difficult decision [45] to migrate because of a need to seek protection from violence [39, 41, 45, 52, 56]. Women were also forced into migration because of a destruction of societal systems, when experiencing a lack of basic resources [38], having a need to protect their family members [39], and because they longed for peace and freedom [39]. Forced migration entailed an involuntary need to give up normal contexts [45] and personal belongings [38, 40, 51]. Social networks helped in the decision making to emigrate [38]. Some women needed to persuade family members to emigrate with them [45].

“The decision to migrate was mostly a joint family agreement. The primary focus was the future of their children and the wellbeing of the larger family.” [39]

“We didn’t want to leave the country. My mom, my kids, we weren’t going to leave... But we were scared for our kids” (Direct quote from a participant) [45]

##### Positive effects related to exposure

While the hardships experienced by women were evident, some reports also describe positive effects of being exposed to armed conflict and/or forced migration. Gaining expanded responsibilities within the family was described, leading to empowerment, greater independence, and a capacity to challenge traditional gender roles [46, 48, 52, 62]. Another positive effect was strengthened relationships with family members and others in similar positions [47, 48, 54].

“Across all of the four research locations, participants described how, during the war, women took responsibility for their families and became less dependent on men. This continued after the war, and women became more confident and more willing to challenge their partners.” [62]

”I learned that I am strong, that I can live and cope with this crazy reality, that I have the courage and strength to cope in any future situation. No matter what happens I will manage by myself. I have much more trust in myself now and this is a result of living here and coping with everything. I also learned to be more flexible, to enjoy my children, not to “drive them to the wall,” because I am aware that we have to live and enjoy the present. No one knows what will happen tomorrow.” (Direct quote from a participant) [48]

#### Theme 2: Health-related consequences when confronted by a complex and demanding situation

##### Psychological distress during armed conflict and forced migration

Having to migrate through illegal or legal routes and to live in displacement involved significant psychological distress, fears, and uncertainties [43, 45–48, 56, 59]. Women were reminded of the significant dangers to their lives when witnessing the death of others [53] and they needed to take responsibility for others (including children) during migration, leading to significant psychological distress [45, 51]. Being exposed to armed conflict and being forced to migrate involved a loss of identity, difficulties accepting their new identity, challenges when trying to adjust to the new context, and feeling grief or emptiness when missing or longing for their previous life [43–46, 52, 56]. Women living in refugee camps perceived the camps as unsafe and inhospitable with poor living conditions, associated with suffering [42, 43, 45, 55, 59] including anxiety, psychological distress, and fears [56, 59], intensified when the camp was close to armed conflicts [41].

”Women feared the general atmosphere in the camp and found it difficult to witness other refugees’ miseries” [45]

”When I slept, I sometimes saw fighting and yelling and I would wake up with a weak state of mind. I was scared that these things will come true, I was scared that these things will happen. You never knew if you're safe there [in Syria] [scared tone while crying]”. (Direct quote from a participant) [56]

##### Being exposed to risks during pregnancy and childbirth

Being pregnant and giving birth involved a particularly vulnerable position, including being exposed to physical and sexual violence resulting in serious consequences for the health of the pregnancy and expected child. Thus, women in these positions felt unsafe and needed to take precautions [55]. Women in forced migration also encountered considerable challenges during and after childbirth, with limited possibilities of accessing quality intrapartum and postpartum care, and did not have the chance to adequately recuperate after childbirth [39, 51]. Some were forced to discontinue breastfeeding and experienced difficulties providing nutrition for their children [39, 51].

”Women also cited direct impacts of insecurity on pregnancy, as a few women openly spoke about 3 instances of seeing (or experiencing) a pregnant woman being beaten by a security guard, staff member, or policeman at the ration center or food distribution center. In all of the accounts of these instances, the pregnancy ended in a miscarriage, still birth, or neonatal death.” [55]

“They [the militia men] abused me. [...] They think that if someone happens to see the genital organs of a married woman, the woman has to be stoned – stoned to death. [...] It was just after the delivery I escaped. I had recently given birth.” (Direct quote from a participant) [39]

##### Being exposed to violence and discrimination as a woman

Women were exposed to intensified violence in public and private settings [38–40, 45, 46, 50, 54, 55, 61, 62, 62], perpetrated by the regime, military, and civilians [39, 40, 45]. Armed conflict and forced migration involved a significant risk of being exposed to violence and threats within the immediate and extended family. It also involved a significant restriction of women’s lives and freedom [44, 46, 49], as a result of strengthened patriarchal structures and men's reactions to the situation [50, 55, 61]. Exposure to violence resulted in women experiencing fear, panic, insecurity, grief, and physical consequences [37, 54, 55]. When her husband died, women were left without a formal protector within the family, exposing her to further danger [50, 51]. Women were also victims of forced marriage as a result of war [39, 50, 62].

”Eight of the women reported emotional abuse from their husbands prior to the war. Of those, five reported that the abuse became physical after the war had begun. The women who had been physically abused prior to the war reported an increase in frequency and intensity after the war began” [61]

”My husband hit me for the first time during the war time. I have never expected him to do so or even to be mad with me ever before the onset of war.” (Direct quote from a participant) [61]

During armed conflicts and forced migration, women and their daughters were at high risk of being subjected to sexual violence, including coercion, repeated rapes, and sexual harassment [39–41, 44, 46, 53, 55, 57–59, 61]. Soldiers and civilians used rape to humiliate and spread fear [40, 58, 59]. Pregnant women were considered particularly exposed to the risk of being raped [55]. As a strategy to reduce the risk of being subjected to sexual violence, women were isolated from the outside world by others such as their husbands or decided to isolate themselves from public spaces and regular activities [38, 39, 42, 55, 61]. When exposed to sexual violence, women encountered a culture of silence, stigma and social exclusion [39, 40, 42, 44, 46, 55, 58]. They also had limited opportunities for induced abortion and legal support [39].

“Fatuma and other women from her village were raped by postpubescent in front of the members of their community, in the presence of their own husbands and children” [58]

”These boys they were my neighbours. I remember them as young boys when I got married. One day he [the rapist] came to my house during the war and asked me to show him all the rooms in the house, and my son was playing in the garden when all of a sudden he took a knife and put it under my neck and asked me if I wanted to do it there by my own will or not, and at that point I knew exactly what would happen. He beat me so I could not breathe, and he kicked me in my stomach. I lost consciousness, and when I regained consciousness he raped me and there was blood all over. When he saw what happened, he just left me alone. He went out and asked the two soldiers that were in front of the house if they wanted to come up and rape me too.” (Direct quote from a participant) [40]

##### Lack of adequate healthcare services

Armed conflict affected the accessibility of healthcare services by introducing a number of structural barriers, including an unstable financial situation, a lack of available services and medications, and service’s difficulties to meet the needs for care within the population [55, 56, 61]. Stigma around mental illness and women's own mental health prevented them from accessing mental health services [56]. Women also had difficulties accessing adequate care during pregnancy and childbirth, involving significant risk and anxiety [45, 55]. When accessing healthcare services, some experienced a disrespectful and unethical treatment from professionals [55].

“Barriers to obtaining health services contained internal and external aspects ranging from personal to interpersonal to socio-economic barriers.” [56]

”Being a refugee is very hard and after all being a woman, it being a difficult life is something obvious. When I was about to give birth, I did not get any ambulance. No ambulance comes here to get you unless you go to the highway and when we get to the high way, we have to wait for some hours for them to come.” (Direct quote from a participant) [55]

##### Lack of social support

Women described a lack of social support from relatives [52] as well as a lack of social support through governmental organizations [49], humanitarian organizations [38, 46, 56], and religious institutions [44]. Changed relationships and a lack of social support contributed to alienation and social exclusion [42, 47], as well as feelings of shame, guilt, loneliness and hopelessness [44, 46]. Staff in the refugee camp lacked the resources to offer support and sometimes posed a threat to women, with violent outcomes [55].

”Camp staff and security personnel contribute in 2 ways to feelings of insecurity within public spaces, by direct harmful actions towards refugees and by the lack of action taken for refugee complaints.” [55]

#### Theme 3: Resources and strategies that enhance resilience

##### Social support and family life

Social networks were important before, during and after forced migration [39, 41, 42, 46, 49, 52, 54, 59], involving the exchange of instrumental [39, 59], informational [52], and emotional support; including peer support between women in similar situations [37, 46, 54, 59]. Family relationships provided psychological relief and reduced psychological distress [37, 43, 46, 49, 54]. Women drew strength from family members to endure challenges, kept the family together [52], and sought support from them [46, 49, 52, 54, 56, 59]. Motherhood was described by women as a source of resilience and agency [39, 41, 46, 47, 49, 51, 53, 54, 59, 60]. When women were placed in certain situations that could involve increased vulnerability, including pregnancy and childbirth, social support was crucial [39].

“Women mentioned social and family ties as vital resources for coping with traumatic realities. Family and friends were considered by women as resources for coping with systematic violence and structural discrimination.” [37]

“The only support you can have is like if you can talk to your sister or to your auntie about it, how you feel, that’s the only counselling, you know.” (Direct quote from a participant) [46]

##### Utilizing internal resources and strategies

Women tried to maintain daily life and their safety by living day by day [37, 39, 48, 49, 54]. They kept hoping for a better future [46, 49, 52–54, 56, 57, 59], for example through religion and faith [39, 41–43, 46, 47, 49, 52, 54, 56, 59], through education and work [47], or by the use of play, humor and optimism [47, 48]. Further coping mechanisms involved stopping visiting imprisoned relatives [57], becoming violent towards their children, developing self-harming behaviors [42], or succumbing to passivity and resignation [48, 59]. Women hid their emotions [51], were vigilant [52], and pretended to not understand languages [60]. They also showed ingenuity to make men less violent [38, 42, 59], protect themselves and their loved ones [51, 59], and maintain mental stability [41]. Through creativity and by showing resistance, women took control of life by finding ways to support themselves [41, 46, 51, 52, 59] and created safe places to stay [39, 48, 55]. When needed, they restarted their lives [54], and engaged in political resistance, which was empowering [48, 54].

“At the same time, despite the ongoing and extreme living conditions, women described continuous attempts to normalize their daily routines in an environment perceived as insecure and threatening. They described themselves as competent in mastering how to cope with ongoing occupation and political oppression” [37]

”The growth of faith in our hearts and our religion. We had a strong faith, we understood that our destiny is in the hands of a single creator and nobody can do anything but him.” (Direct quote from a participant) [54]

## Discussion

The aim of this systematic review of empirical qualitative research was to provide a comprehensive perspective on adult women’s experiences of armed conflicts and forced migration, focusing on those in or from countries in the Middle East, Balkans, or Africa. The results portray a highly demanding and complex situation for women, who suffer of changed living conditions, gender-based violence, and significant health-related consequences. The results illustrate a range of interpersonal and intrapersonal resources and strategies utilized by women to enhance their strength and resilience when faced with challenging circumstances.

In part, the findings on violence and health-related consequences confirm what has been widely established through a breadth of research investigating resettling refugees in general. Armed conflicts and forced migration are highly challenging processes that entail significant health-related consequences among asylum seekers and refugees, including high prevalence of depression, anxiety, and post-traumatic stress [63–65]. In addition to the challenges experienced by refugees regardless of gender, our findings highlights numerous stressors women encounter specifically.

Evidence syntheses through literature reviews have shown that refugee women in resettlement experience damaging effects related to significant disparities in access to healthcare services [17, 66–68] and have worse health-related outcomes than non-immigrant women [69, 70]. Our findings complement these reviews by illustrating the impactful challenges and consequences women face before arriving in the host country, including considerable risks and consequences related to patriarchal structures in society, violence against women (including sexual violence), pregnancy, and childbirth. Undoubtedly, the violent and stressful stories provided through the reports call attention to the need to improve the safety and health of women experiencing armed conflicts and forced migration. The exposure to violence and health-related consequences targeting and affecting women expanded beyond the immediate conflict zone, also involving forced migration through severe circumstances with poor access to healthcare services and when staying in refugee camps. We did not identify any conclusive qualitative evidence concerning women’s experiences of torture, highlighting a need for more research exploring this topic in detail. Taken together, there is a need for societal-level changes increasing the health and safety of women living in settings with armed conflicts and those being forced to migrate. Our findings illustrate the many significant challenges women face in these dire situations; including unsafe housings, inaccessible healthcare services, a lack of basic resources such as food, and insufficient support from governmental as well as non-governmental organizations. Considering the violent and dangerous circumstances depicted in the included reports, women need substantial protection and improved living conditions in these settings. There is a convincing need of rigorous research designing and implementing interventions aiming to support these women as they live in conflict zones and when they are forced to emigrate.

An important finding is the numerous interpersonal and intrapersonal resources women utilized to find strength and endure the hardships they experienced. Having social support contributed to enhanced resilience, while a lack of social support contributed to psychological distress and social exclusion. Previous studies suggest that social support can have a protective effect in war and may improve mental health in refugees [71–73]. Some studies have also shown promising results of group-based psychosocial interventions [74–76]. According to our findings, women engaged in social support that involved emotional, informational, and instrumental support. Interestingly, women utilized peer support, meaning they provided and received support from women in similar situations as themselves. Indeed, peer support interventions among refugee women have been suggested as relevant and effective interventions, potentially resulting in reduced social exclusion and mental health burdens [77]. Besides social support, women also drew strength from faith and religion to endure the hardships they encounter during armed conflicts and forced migration. In line with these findings, studies have illustrated that religion is utilized by many refugees to facilitate coping with psychological distress [73, 78, 79]. Taken together, our review portrays the individual potential positive effects that social support and faith can have for refugees. Clinicians supporting these women should assess their individual resilience-building resources and explore how women can be empowered, including social support and faith. Our findings also highlight the need for more experimental clinical research aiming to enhance resilience and promote mental health among these women through social support and internal resources.

There are methodological considerations of this study that needs to be taken into consideration. The systematic searches were performed in three databases and records were independently screened by two of the authors. Conflicts were settled through discussions. We conducted manual screenings to identify further reports not produced through the systematic searches in the utilized databases. Nevertheless, we cannot disregard the potential risk that some reports could have been wrongfully excluded during the screening process or that other reports would have been identified if more databases, and/or additional search terms, had been utilized. Importantly, we only used “woman” as a singular search term related to the gender of interest. This is a methodological limitation that needs to be considered when interpreting our findings. As with all qualitative analyses, the thematic synthesis herein is intrinsically biased by the views and preconceptions by the analysts. To strive towards neutrality towards the data, several authors were involved throughout the thematization process. The two authors responsible for the primary analysis have clinical experience of supporting refugee women during resettlement (one as a psychologist and the other as a physiotherapist). However, we cannot dismiss the possibility that some nuances and perspectives were lost during the analysis. Public involvement utilizing women with lived experiences of armed conflicts and/or forced migration has the potential to enhance the relevance of the aims and findings of research. Unfortunately, no public involvement was utilized in this review and we acknowledge that this is a limitation that could have impacted the findings. We encourage researchers to involve these women when conducting future reviews and empirical studies. To reach clinically applicable results about a defined population, we aimed to include reports with participants from the Middle East, Balkans, and Africa. Most of the reports recruited participants from Africa, while fewer reports had participants from the Middle East or the Balkans. We acknowledge that other parts of the world host refugees with other countries of origin than those represented herein. Displacement is a changing phenomenon over time, and it is probable that other countries of origin among displaced persons will emerge in the future. Thus, the transferability of the results is limited in regard to geographical settings and this could change over time. Guided by the paucity of syntheses on qualitative research exploring experiences among women before resettlement, this review did not focus on post-migration experiences. We encourage researchers to conduct additional reviews that will complement the findings herein.

## Conclusion

When experiencing armed conflicts and forced migration, women face significant challenges related to changed living conditions, gender-based violence, and health-related consequences. Consistently, women are targets of severe structural and personal violence, while lacking access to even the most basic healthcare services. Societal changes are needed to improve the protection and rights of women in these settings. Despite facing considerable trauma, these women display considerable resilience and endurance by finding strength through social support and internal resources. Synthesized qualitative research illustrates that women value social support, including peer support. Peer support is a promising intervention that needs to be evaluated in future experimental studies.

## Supplementary Information

**Additional file 1.** The ENTREQ checklist.

## Data Availability

Not applicable.
